# High Concentrations of Angiopoietin-Like Protein 4 Detected in Serum from Patients with Rheumatoid Arthritis Can Be Explained by Non-Specific Antibody Reactivity

**DOI:** 10.1371/journal.pone.0168922

**Published:** 2017-01-20

**Authors:** Elena Makoveichuk, Toralph Ruge, Solveig Nilsson, Anna Södergren, Gunilla Olivecrona

**Affiliations:** 1 Department of Medical Biosciences/Physiological Chemistry, Umeå University, Umeå, Sweden; 2 Department of Surgery and Perioperative Sciences/Acute Medicine, Umeå University, Umeå Sweden; 3 Department of Public Health and Clinical Medicine/Rheumatology, Umeå University, Umeå, Sweden; Centre National de la Recherche Scientifique, FRANCE

## Abstract

Angiopoietin-like protein 4 (ANGPTL4) is suggested to be a master regulator of plasma triglyceride metabolism. Our aim was to study whether the previously reported high levels of ANGPTL4 detected in serum from patients with rheumatoid arthritis (RA) by ELISA was due to any specific molecular form of this protein (oligomers, monomers or fragments). ANGPTL4 levels were first determined in serum from 68 RA patients and 43 age and sex matched control subjects and the mean values differed by a factor of 5.0. Then, ANGPTL4 was analyzed after size exclusion chromatography (SEC) of serum samples. With serum from one of the RA patients with high levels of ANGPTL4, the dominant reactivity was found in fractions corresponding to high-molecular weight proteins. In addition, a minor peak of reactivity eluting late from the column was found both in the patient and in controls. By the use of HeteroBlock®, and by careful selection of antibodies, we documented non-specific reactions for ANGPTL4 in 39% of samples from the RA patients, most likely due to cross-reactivity of the antibodies with rheumatoid factor (RF). The corresponding figure for control subjects was 6.3%. After corrections for non-specific reactions, the mean level of ANGPTL4 in serum from RA patients was still significantly higher than in control individuals (mean levels were 101±62 and 67±39 ng/ml respectively, P = 0.02). We re-analyzed samples from our previously published studies on ANGPL4 levels in patients on hemodialysis and patients with diabetes type 2. These samples did not show false positive reactions. The levels of ANGPTL4 were comparable to those detected previously.

## Introduction

Angiopoietin-like protein 4 (ANPTL4) belongs to the family of angiopoietin-like proteins [1, 2]. The C-terminal part of ANGPTL4 has anti-angiogenic properties [3], while the N-terminal part inactivates lipoprotein lipase (LPL), the key enzyme for metabolism of plasma triacylglycerols (TG) [2, 4, 5]. ANGPTL4 is highly expressed in liver [6] and is detected in blood [7]. ANGPTL4 expression is stimulated by peroxisome-proliferator activated receptors (PPARs) that in turn are activated by fatty acids [7]. Therefore ANGPTL4 is a major suppressor of LPL activity in adipose tissue under fasting conditions [2, 5, 8]. ANGPTL4 also controls LPL activity in heart and skeletal muscle in order to prevent excessive lipid uptake [9, 10].

Genetic defects of ANGPTL4 in humans are associated with lower levels of plasma TG [11]. The metabolic role of ANGPTL4 in blood and its ability to inactivate LPL remains, however, unclear [2, 12]. For unknown reasons the concentrations of ANGPTL4 in some plasma samples have been found to be 10–20 fold higher than the values obtained in most other samples from comparable groups of subjects [13–16]. An unusually large fraction of samples from patients with rheumatoid arthritis (RA) (37% of all sera analyzed) were reported to have levels of ANGPTL4 higher than 170 ng/ml [15]. The authors proposed that the concentration of ANGPTL4 in serum could be used as a novel marker for bone destruction in RA.

ANGPTL4 is known to appear in several molecular forms like monomers, dimers and oligomers and the protein may also be cleaved between the two domains to fragments of about half the molecular weight of the full length protein [17]. The primary aim of our study was to investigate in what forms ANGPTL4 are present in human plasma. In particular, we wanted to investigate which of these forms could explain the high levels of ANGPTL4 in blood from RA patients.

## Material and Methods

### Patients and controls

Blood samples were obtained within a structured program on patients with early RA for prospective analysis of development of co-morbidity using the nationwide Swedish Rheumatoid Arthritis Registry [18–20]. In short, all eligible patients with newly diagnosed RA (ACR criteria) [21] are continuously enrolled into the register. Between the years 2000 and 2004 all newly diagnosed patients with RA under the age of 60 years were included into a study on the progression of atherosclerosis [18–20]. Of these patients, 68 were followed up after five years, and data in the present study are from that follow up [20]. Forty three age- and sex matched controls were also included. All patients were examined clinically at inclusion into the study and regularly thereafter. The number of swollen and tender joints (28 joint count) and the patient’s global assessments were registered, and a disease activity score (DAS28) including the erythrocyte sedimentation rate (ESR), was calculated [22]. All individuals gave their written consent in accordance with the Declaration of Helsinki. The study was approved by the Regional ethics committee of Umeå University, Umeå, Sweden.

### Blood sampling and analyses

For the present study all patients and controls donated blood samples at the time of follow up five years after inclusion. No dietary restrictions were made for these samples. For analyses of glucose and lipids in blood, a separate blood sample was collected after regular overnight fasting. Serum was separated and stored at -80°C. ANGPTL4 was measured in sera from 68 RA patients and 43 matched controls using the DuoSet ELISA Development kits (DY3485) from R&D Systems (Abingdon, UK) with four different lots of detection antibodies–XPQ0109091 (lot I), XPQ0109011 (lot II), XPQ0310101 (lot III) and XPQ0413021 (lot IV). The ELISA procedure was modified as previously described [23]. In some cases analyses were made using the un-modified ELISA procedure recommended by the supplier, or using sandwich ELISAs with different combinations of capture and detection antibodies, as specified in the Results section. The ELISAs were run with serial dilutions, and with both control and RA-samples together on the same plate. For each plate internal controls were also included. The intra-day and inter-day coefficients of variation for ELISAs did not exceed 14% and 7%, respectively. Some stored serum samples from subjects with insulin resistance and type 2 diabetes mellitus, participating in a previous study of effects of hyperinsulinemia on the levels of ANGPTL4, were used for comparison [24]. In addition we re-analyzed samples from a previous study of ANGPTL4 in patients on chronic hemodialysis (CHD) [25]. Pooled normal human serum was used for isolation of very low density lipoproteins (VLDL) by ultracentrifugation [26].

For studies of the specificity of the ELISA, a recombinant, His-tagged N-terminal fragment spanning residues 26–229 of human ANGPTL4, as well as the full length molecule of human ANGPTL4, both expressed in *E*. *coli*, were kindly provided by Dr. Mikael Larsson (Umeå, Sweden). The recombinant C-terminal fragment of human ANGPTL4 was purchased from AdipoGen (AG-40A-0070). All forms of ANGPTL4 showed high reactivity in the ELISA. We verified that the immunoreactivities were not blocked by the presence of normal human serum or isolated human VLDL (corresponding to 2 mg TG/ml), and was not changed by denaturation of the proteins in 1% sodium dodecylsulfate (SDS). We concluded that different forms of the ANGPTL4 protein could be detected in serum by the modified ELISA (data not shown).

We used size exclusion chromatography (SEC) to study the distribution of different forms of ANGPTL4 in plasma and to analyze their potential association with plasma lipoproteins. For this, 0.4 ml of fresh serum was applied to a Superose® 6 HR 10/30 column (Pharmacia Biotech) equilibrated in phosphate buffered saline (PBS, 10 mM phosphate, 0.16 M NaCl, pH 7.4), containing 10 mM EDTA and eluted on an Äkta Purifier (GE Healthcare) at a flow rate of 0.5 ml/min. The protein concentration was monitored by continuous measurement of absorbance at 280 nm. Fractions (0.6 ml) were collected on ice, diluted with 0.1 volumes of a solution containing 10% (w/v) bovine serum albumin and 1% (v/v) Tween20, and were then immediately used for analysis of ANGPTL4 by the ELISA. Total cholesterol (TC) and TG were detected in serum before SEC and in column fractions using Cholesterol CHOD-PAP and Triglycerides/Glycerol Blanked Cobas® reagents, respectively (Roche Diagnostics GmbH, Mannheim, Germany). The distribution pattern for ANGPTL4 reactivity in serum from healthy donors was found to be independent on whether blood was drawn after fasting overnight or in the postprandial state, or whether the serum was treated with the lipase inhibitor tetrahydrolipstatin or not before SEC (data not shown).

Rheumatoid factor (RF), C-reactive protein (CRP) and erythrocyte sedimentation rate (ESR) were measured according to routine methodology for clinical laboratories (Waaler-Rose for RF). Intima and media thickness (IMT) was measured as described [18, 20]. Serum concentrations of cholesterol, TG and high-density lipoprotein (HDL) in blood samples after an overnight fast were measured using routine methods. The LPL protein mass was detected as described previously [27], but using purified bovine LPL as standard [28].

### Statistics

Differences in variables between patients with RA and matched controls were analysed using simple conditional logistic regression analyses. Comparisons within the RA patient group were performed using the Mann-Whitney U-test. Simple linear regression analyses were used to identify variables associated with ANGPTL4. P-values <0.05 were considered as statistically significant. All statistical calculations were made using SPSS 18.0 (SPSS Inc, Chicago, US).

## Results

Using the commercial ELISA-kit for ANGPTL4 provided by R&D Systems we found several-fold higher amounts of ANGPTL4 in serum from RA patients compared to their controls (mean values were 413 and 82 ng/ml, respectively). In twenty eight samples from the RA group (41.2% of the group), compared to only in two from the control group (4.3%), the concentration of ANGPTL4 exceeded 150 ng/ml. Very high levels of ANGPTL4 reactivity, ranging from 1000 to 4000 ng/ml, were detected in eight cases in the RA group (11.8%), but none with such high levels was found in the control group. Analyses of ANGPTL4 using the original ELISA protocol or the modified procedure from our laboratory [23], gave similar results (data not shown).

**Fig 1:**
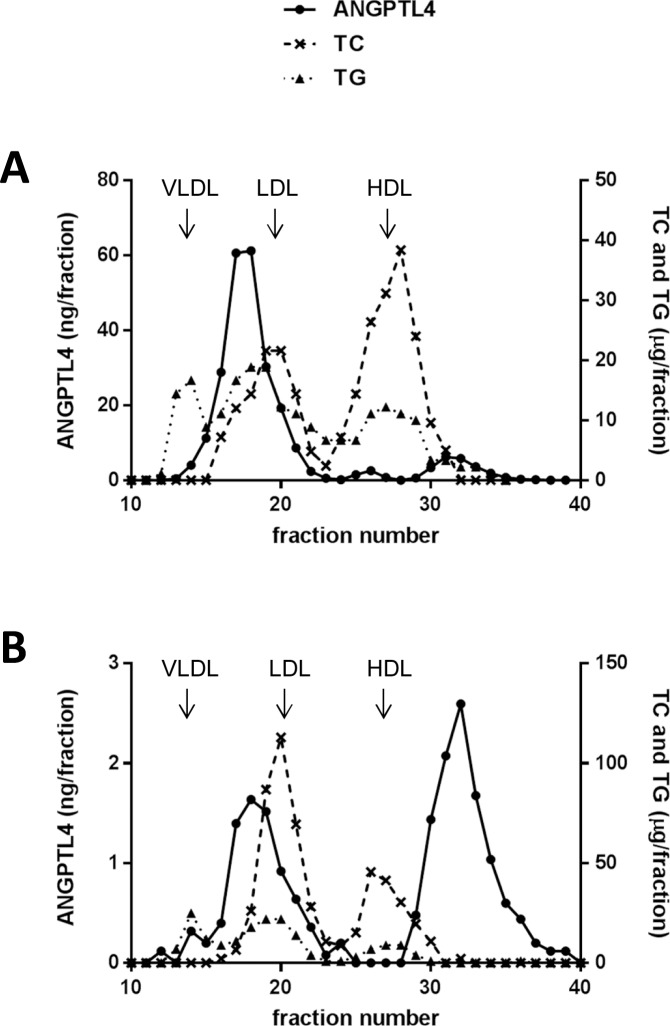
Analysis of the distribution of ANGPTL4 immunoreactivity after separation of human serum by SEC. Serum from one patient with rheumatoid arthritis (RA) and a high level of ANGPTL4 reactivity (A) or from a healthy age and sex matched donor (B) was subjected to chromatography on a Superose® 6HR column. ANGPTL4 immunoreactivity in the fractions was measured by the ELISA using antibody XPQ0109091 (lot I) for detection (*solid* lines). Concentrations of total cholesterol (TC, *dashed* lines) and triacylglycerol (TG, *dotted*-lines) were measured in the fractions to localize the elution positions of VLDL, LDL and HDL (indicated by arrows). The highest levels of ANGPTL4 reactivity were found in fractions obtained from RA-serum and eluted in a position between that of VLDL and LDL.

Next we used size exclusion chromatography (SEC) of serum to separate different molecular forms of ANGPTL4 and to study if any form was associated with plasma lipoproteins. Serum from a patient with a high level of ANGPTL4 (837 ng/ml) was selected for the separation shown in Fig 1A. The highest levels of ANGPTL4 reactivity were eluted in a position between that of VLDL and low density lipoprotein (LDL). There was a second, small peak of ANGPTL4 reactivity eluting after the high density lipoprotein (HDL) (Fig 1A). A similar distribution of ANGPTL4 reactivity was found in serum from normal, healthy donors (with levels of ANGPTL4 between 21 and 49 ng/ml), but the first peak was then small and the main peak was that one eluting after HDL (Fig 1B, notice the different scale on the y-axes between panels A and B).

The use of a reagent called HeteroBlock® (Omega Biologicals, Inc., Bozeman, MT), designed to eliminate interference caused by heterophilic antibodies in sandwich immunoassays, is recommended to minimize false positive reactions in ELISA analysis in patients with RF. To investigate the possibility that we detected non-specific reactions in the ELISA for ANGPTL4 in sera from patients with RA, we added HeteroBlock® to the SEC fractions. When added in high concentration, HeteroBlock reduced the immunoreaction for ANGPTL4 in fractions from the first peak, while the reaction in the second peak was unaffected (Table 1). In addition, HeteroBlock® added at 50 μg/ml eliminated most of the high immunoreactivity for ANGPTL4 in sera from patients with RA (Table 1). From this we concluded that the high reactivity for ANGPTL4 in serum from RA patients was most likely due to a false, non-specific reaction with RF/IgM.

**Table 1:** HeteroBlock® reduced the immunoreactivity in the ANGPTL4 ELISA indicating false positive reactions in sera and chromatography fractions.

	ANGPTL4 concentrations detected with lot I or lot II (ng/ml)		
	without HeteroBlock®	with HeteroBlock®	
Group		50 μg/ml	150 μg/ml
RA patients (n = 3)	716	235	270
	937	49	
	1735	174	
Non-RA-subjects (n = 6)	82^a^	60 ^a^	
	120	106	
	210 ^a^	186 ^a^	
	418^b^	357^b^	339^b^
	665	44	
	1261^a^	1134^a^	
SEC, peak 1 (n = 4)	11.5	9.0	
	59.9	53.3	
	5.2		3.6
	55.4		23.4
SEC, peak 2 (n = 2)	10.7	10.7	10.3
	43.3	42.5	42.7

^a^ Subjects from a previous study on ANGPTL4 in patients with insulin resistance and type 2 diabetes mellitus.

^b^ Subject with a high level of ANGPTL4 from a previous study of patients on chronic hemodialysis (CHD).

To investigate whether the capture antibody or the detection antibody from the DuoSet ELISA Development kit (DY3485) was responsible for the false positive reaction for ANGPTL4, we performed ELISAs on serum samples using the same capture antibody but with four different lots of detection antibody (see Methods section). With two of them (lots I and II), high amounts of ANGPTL4 were detected in sera from RA patients, while with the other two (lots III and IV) the reactivity was in the normal range (Table 2). For comparison we used serum samples from eleven patients on chronic hemodialysis with elevated amounts of ANGPTL4 from our previous study [25]. For these samples there were no differences in reactivity with the different antibodies.

**Table 2:** Verification of false detections of ANGPTL4 in sera and SEC-fractions.

	ANGPTL4 concentrations (ng/ml) obtained with different lots of detection antibody^a^		
Group	Lot I or II	Lot III or IV	Cases with false positive detection^b^ (% of all samples)
RA patients (n^c^ = 57)	16–3930^d^	32–364^d^	39
Controls (n^c^ = 16)	18–725	41–137	6.3
CHD patients (n^c^ = 11)	131–435	171–328	0.0
SEC, peak 1 (n^e^ = 26)	0.3–170	0–3.7	83
SEC, peak 2 (n^f^ = 13)	1.1–20.0	3.0–19.6	0.0

RA, rheumatoid arthritis; CHD, chronic hemodialysis.

^a^ Detection antibody from DuoSet ELISA Development kit (DY3485, R&D Systems).

^b^ Based on the strong interaction with HRP-conjugated goat polyclonal antibody against Fc-fragment of mouse IgG (Sigma-Aldrich, A2554).

^c^ Number of individuals.

^d^ The ranges of all values are presented.

^e^ Total number of SEC-fractions analyzed from runs of 5 individual serum samples.

^f^ Total number of SEC-fractions analyzed from runs of 4 individual serum samples.

Antibodies from lots I and II showed immunoreactivity with the early peak fractions after SEC of serum samples, while antibodies from lots III and IV showed little or no reactivity with this peak (Table 2). ANGPTL4 immunoreactivity in fractions from the second peak was independent of the lot of antibodies used (Table 2). We concluded that the ELISA with detection antibody from lots III and IV demonstrated the true elution position of ANGPTL4 after SEC of serum samples, while lots I and II showed cross-reactivity with RF that eluted early form the column.

To demonstrate that the capture antibody was also involved in the false detection of ANGPTL4 by the DY3485 kit, we had to change the system for detection. It was not possible to investigate the specificity of capture antibody with the one provided with the kit (with biotinylated antibody followed by HRP-conjugated streptavidin). We performed the ELISA using the same capture antibody, but for detection we used a mouse polyclonal antibody against ANGPTL4 (Abnova, H00051129-B01P), as a primary antibody, followed by HRP-conjugated goat polyclonal antibody against the Fc-fragment of mouse IgG (Sigma-Aldrich, A2554). The results from the ANGPTL4 analyses were similar to those obtained with detection antibody lots I and II from DY3485 kit for the same samples (data not shown). Incubations without primary antibody were run in parallel. We found that the sera from RA patients with non-specific reaction for ANGPTL4 (that could be eliminated by HeteroBlock®), as well as the SEC fractions corresponding to the first peak of ANGPTL4 reactivity, had reacted strongly with the goat anti-mouse Fc-fragment antibody (Table 2, right column). By the same procedure we analyzed serum samples from 57 patients with RA and 16 corresponding control subjects. In the RA group the binding of the A2554 antibody was very strong in twenty two cases, but only in one case in the control group (Table 2). Seventy seven percent of RA patients with strong binding of the A2554 antibody were also RF positive (RF+, data not shown) and showed the highest levels of serum ANGPTL4 in the ELISA using the detection antibody from lot I. Based on these results we concluded that the capture antibody from DuoSet ELISA Development kit (DY3485, R&D Systems) bound RF and thus was the primary cause for the false positive detection of ANGPTL4.

**Fig 2:**
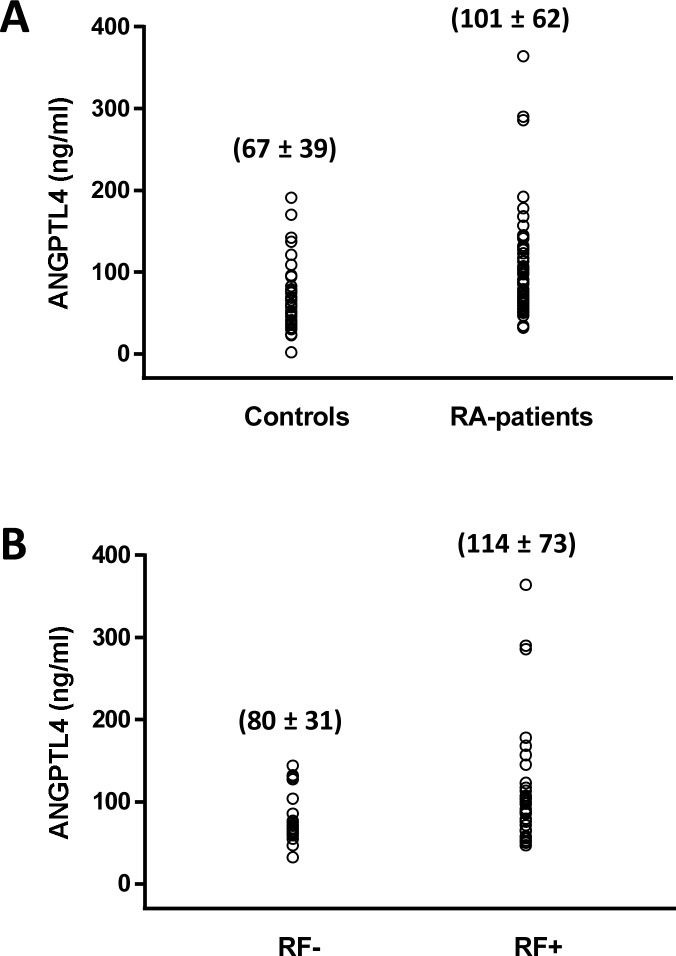
Levels of ANGPTL4 in sera from RA patients and controls after correction for the false positive reactions to rheumatoid factor (RF). (A) ANGPTL4 was measured in sera from 57 patients with rheumatoid arthritis (RA) and 43 matched controls by ELISA using DY3485 kit with lot IV detection antibody. (B) ANGPTL4 was measured as in (A) in sera from RF+ (n = 33) and RF- (n = 20) patients with RA. The means ± SD are indicated.

The non-specific interaction of RF with the capture antibody from DY3485 kit was not seen with lots III and IV of detection antibodies. We re-analyzed the serum samples from 57 patients with RA that had primarily been analyzed with detection antibody from lot I. The mean value obtained with lot I antibodies (443 ng/ml) was 4.5 times higher than the mean value obtained for the same samples with lot IV antibodies (101 ng/ml). For comparison we repeated the measurements of ANGPTL4 in 16 samples from the control group that included two samples with ANGPTL4 concentrations higher 150 ng/ml obtained with lot I antibodies. The ANGPTL4 concentration detected with lot IV antibodies was in both of these samples around 50 ng/ml, while with all the other control samples the detected level of ANGPTL4 was similar with lot I and lot IV antibodies. After excluding the false positive detections, the concentrations of ANGPTL4 in serum of patients with RA remained significantly higher (P = 0.02, logistic regression analyses) in comparison with the control group (Fig 2A). No significant differences for the levels of ANGPTL4 were found between men and women in either of the groups (data not shown). Within the RA group, significantly higher levels of ANGPTL4 (P = 0.04, Mann-Whitney U-test) were detected in serum from RF+ patients than in serum from RF negative (RF-) patients (Fig 2B).

Final results (corrected for false positive reactions) from the RA patients and control subjects were used in linear regression analyses based on data presented in Table 3. No significant associations were found between ANGPTL4 concentrations and any of the variables tested, i.e. age, duration of RA, ESR, DAS28 (measurement of disease activity for patients with RA), IMT (a measurement of atherosclerosis development), blood pressure, body mass index, smoking, previous cardiovascular disease (CVD), diabetes mellitus, plasma TC, TG, LPL protein, HDL or CRP concentrations (Table 4).

**Table 3:** Descriptive data on 57 patients with RA and 43 age- and sex matched controls.

	Patients with RA (n = 57)^a^	Controls (n = 43)^a^	P-value
Age, years	46.6 (10.7)	48.1 (10.9)	
Women, n (%)	49 (86%)	32 (74%)	
Smoking ever, n (%)	36 (63%)	20 (49%)	0.06
Previous CVD, n (%)	6 (11%)	2 (5%)	0.3
RF +, n (%)	33 (58%)		
Disease duation, months	76.2 (7.0)		
ESR, mm/h	16.5 (12.3)		
DAS28	3.2 (1.5)		
IMT, mm	0.57 (0.1)	0.60 (0.1)	0.2
Serum cholesterol, mmol/L	5.3 (1.0)	5.6 (1.1)	0.1
HDL cholesterol, mmol/L	1.6 (0.5)	1.7 (0.5)	0.6
Triglycerides, mmol/L	1.1 (0.5)	1.0 (0.5)	0.4
Blood pressure, systolic, mm Hg	125.8 (14.0)	123.8 (12.0)	0.6
Blood pressure, diastolic, mm Hg	76.6 (7.9)	77.3 (7.1)	0.7
Body mass index	25.3 (4.1)	25.2 (4.2)	0.9
LPL protein, ng/ml	33.9 (11.4)	39.1 (14.1)	0.06
CRP, mg/L	7.1 (5.6)		
Diabetes mellitus, n (%)	2 (4%)	0 (0%)	0.3

CRP, C-reactive protein; CVD, cardiovascular disease; ESR, erythrocyte sedimentation rate; HDL, high density lipoprotein; IMT, intima media thickness.

^a^ Data presented as mean (±SD) when not otherwise stated.

**Table 4:** Simple linear regression analysis in 57 patients with RA with serum levels of ANGPTL4 concentration as dependent variable.

	β	95%CI	p-value
Age	0.85/year	-1.46;1.63	0.91
IMT	292/mm^2^	-1014;1599	0.66
Disease duration of RA	0.32/month	-2.21;2.86	0.80
Systolic blood pressure	-0.34/mmHg	-1.54;0.86	0.57
Diastolic blood pressure	0.41/mmHg	-1.72;2.54	0.70
Body mass index	2.14/unit	-1.91;6.19	0.29
Cholesterol	1.74/ mmolL^-1^	-14.3;17.9	0.83
HDL	-29.5/ mmolL^-1^	-63.9;4.95	0.09
Smoking, ever	-0.18/yes	-34.8;34.4	0.99
Previous CVD	-48.8/yes	-101.7;4.06	0.07
DAS28	-5.57/unit	-25.5;14.4	0.57
CRP	1.74/mgL^-1^	-2.82;6.30	0.44
ESR	0.16/mmH^-1^	-1.18;2.12	0.87
LPL protein	-0.56/ngmL^-1^	-1.44;0.31	0.20
Triglycerides	-3.27/ mmolL^-1^	-37.9;31.3	0.85
Diabetes mellitus	24.4/yes	-67.1;115.9	0.60

CRP, C-reactive protein; CVD, cardiovascular disease; DAS28, disease activity score; ESR, erythrocyte sedimentation rate; HDL, high density lipoprotein IMT, intima media thickness.

## Discussion

Our study emphasizes the importance of using carefully validated antibodies for immunoassays of ANGPTL4 in human plasma. We demonstrate that some of the antibodies that have been used in commercial kits for measurements of ANGPTL4 unfortunately cross-react with rheumatoid factor (RF/IgM). This can probably explain the 9-fold higher levels of ANGPTL4 reported in blood from patients with RA compared to controls, and also the large inter-individual variation of the ANGPTL4 levels (from 24 to 2235 ng/ml) reported in the same study [15]. In some other studies the concentrations of ANGPTL4 were 10 to 20 fold higher in scattered samples compared to the mean concentration obtained for the patients or controls [13, 14, 16]. In most of these studies a commercial ELISA kit for ANGPTL4 (DuoSet ELISA Development kit (DY3485) from R&D Systems) or individual antibodies from R&D Systems were used. Using the same ELISA kit (DY3485) as Swales et al [15] on our group of patients with RA we got similar results as they did. The ANGPTL4 level exceeded 150 ng/ml in 41% of all samples from the RA group, while in the control group only two samples out of 43 had higher levels than 150 ng/ml. After SEC of serum from an RA patient with high level of immunoreactivity for ANGPTL4 we found the highest reactivity in fractions corresponding to proteins of very high molecular weight. Only a minor peak of immunoreactivity was detected in fractions corresponding to the elution position of ANGPTL4.

It is known that the use of ELISA and other immunological methods for analyses of samples from patients with RA requires special care [29]. In multiplex assays of cytokines and chemokines in samples from patients with RA a more than 100-fold amplification of the signal was found to be due to the presence of RF in the samples [30, 31]. The reason is that RF, an autoantibody predominantly of the IgM class [31], is able to interact with the Fc-fragment of normal IgG. A special concern is that RF is also detected in blood samples of 3–5% of a healthy population. This number was increased with older subjects and was also higher in groups of patients with autoimmune or infection diseases [32]. The reagent HeteroBlock® has been developed to prevent or eliminate false positive signals in immunological assays and was shown to be effective in multiplex assay of samples from people with RA [30]. We found that treatment of fractions after the SEC separation of serum from one of the RA patients with HeteroBlock® markedly decreased the immunoreactivity of the first peak in the ANGPTL4 ELISA. Thus, the first peak of ANGPTL4 reactivity, eluting between VLDL and LDL, was most likely due to non-specific binding of the anti-ANGPTL4 antibodies to RF/IgM. We concluded that the high reactivity for ANGPTL4 in serum from RA patients could be explained by a non-specific reaction of the antibodies with RF/IgM. This conclusion was consistent with the early elution position of this reactivity on SEC, indicating a high molecular weight of the antigen.

The ELISA procedure used here, and described earlier [23], is able to detect all forms of ANGPTL4 (monomers, oligomers and fragments) in human serum. After separation of serum samples by SEC and analyses of the fractions in the presence of HeteroBlock® we conclude that ANGPTL4 circulates in blood in a form that elutes after HDL on SEC. Our results differ from those of Mandard et al. [33] who demonstrated that ANGPTL4 is present in human plasma in two forms, a full-length version and truncated one, and that both of them were associated with HDL.

Analyses of ANGPTL4 in sera using DY3485 kits with four different lots of detection antibodies, and using modified procedures for the ELISA, allowed us to conclude that binding of RF/IgM occurred to the capture antibody used in DuoSet ELISA Development kit (DY3485) and was the primary explanation for the non-specific detection of ANGPTL4 by antibodies from lots I and II. In contrast, antibodies from lots III and IV did not bind to RF/IgM and could therefore be used for analyses of ANGPTL4 in sera from patients with RA.

After re-estimation of ANGPTL4 concentrations in the sera from RA patients and control subjects by ELISA with lots III and IV of detection antibody, the levels of serum ANGPTL4 in the RA group were still significantly higher than in the control group. Within the RA group the RF+ patients had significantly higher ANGPTL4 levels than the RF- patients. The inter-individual variations in ANGPTL4 concentrations were rather large, both in the control and RA groups, with the highest values observed for RA patients. The reason for this variation is unknown, but even after exclusion of the highest values, the difference between groups was statistically significant (data not shown). The differences between the groups were, however, not large enough to qualify ANGPTL4 as a useful biomarker of disease activity in RA. In our study, no associations were found between serum ANGPTL4 concentrations and any of parameters analyzed, including CRP. This is in contrast to another study, performed with antibodies from R&D Systems, where positive correlations were found between serum ANGPTL4 and several different parameters related to inflammation, among them CRP [16]. We found, however, that the high levels in many cases were due to non-specific reactivity of the commercial antibodies with rheumatoid factor (RF) that is present at different levels in blood from patients with RA. RF is usually a variant of IgM and also appears in blood of a few percent of otherwise healthy people [32]. Therefore, this non-specific reaction had to be carefully investigated in order to allow analyses of ANGPTL4 in human plasma without disturbing cross-reactivity. A limitation of our study is that we have investigated only one of the currently commercially available ELISA's for the study of ANGPTL4 in blood.

We had previously reported that patients undergoing chronic hemodialysis (CHD), had elevated levels of ANGPTL4 in blood [25]. In light of the problems with non-specific immunoreactions in the ELISA for ANGPTL4, some of the old samples were re-analyzed under our new, carefully controlled conditions. We found that none of the patient samples showed falsely elevated levels. The same conclusion was made from re-analyses of samples from pre-diabetic and diabetic individuals undergoing a study with hyperinsulinemic, euglycemic insulin clamps [24].

In conclusion, antibodies that are used for studies on ANGPTL4 needs to be carefully checked for their specificity. This turned out to be especially important when samples from patients with RA, or likely also with some other cause for inflammation, are analyzed.
